# Tunneled Island Flaps for the Reconstruction of Nasal Defects: A 21-Case Series

**DOI:** 10.3390/jcm12237473

**Published:** 2023-12-02

**Authors:** Fernando Moro-Bolado, Marcos Carmona-Rodríguez, Omar Alwattar-Ceballos, Laura Martínez-Montalvo, María Rogel-Vence, Prado Sánchez-Caminero, Guillermo Romero-Aguilera

**Affiliations:** 1 Department of Dermatology, Marqués de Valdecilla University Hospital, 39008 Santander, Spain; 2 Department of Dermatology, Ciudad Real University General Hospital, 13005 Ciudad Real, Spain; mcarmonarodriguez@sescam.jccm.es (M.C.-R.); oalwattar@sescam.jccm.es (O.A.-C.); lmmontalvo@sescam.jccm.es (L.M.-M.); madelsc@sescam.jccm.es (P.S.-C.); guillermor@sescam.jccm.es (G.R.-A.); 3 Department of Dermatology, Virgen del Mar University Hospital, 28016 Madrid, Spain; dramariarogel@gmail.com

**Keywords:** dermatologic surgery, melolabial island flap, paramedian forehead island flap, nasal pyramid surgery, skin cancer

## Abstract

(1) Background: The reconstruction of cutaneous defects following surgical procedures in the nasal pyramid presents a challenge due to the limited amount of available tissue. In cases of larger defects, skin from adjacent units is used. Traditionally, two-stage surgical flaps have been employed for reconstructing these defects. Tunnelized island flaps allow for the one-stage surgical reconstruction of nasal pyramid defects, using tissue from the forehead or cheek for the flap. (2) Methods: Descriptive retrospective study of 21 consecutive patients who underwent surgery for defects on the nasal pyramid using tunnelized island flaps. (3) Results: Surgical reconstruction was performed in 21 patients with basal cell carcinomas, 14 of them using the melolabial island flap and 7 using the paramedian forehead island flap. In all cases except one, clear histological margins were obtained. Immediate complications were mild and minor. It is worth noting the trapdoor effect complication, which improved over time in most cases, resulting in a satisfactory cosmetic outcome. No tumor recurrences were observed during an average follow-up period of 17.7 months. (4) Conclusions: Tunnelized island flaps allow for single-stage reconstruction of nasal pyramid defects, yielding excellent cosmetic results by utilizing adjacent skin. This procedure demands a certain level of skill but is associated with minimal complications, making it a valuable alternative in reconstructive dermatological surgery.

## 1. Introduction

Cutaneous defects following dermato-oncology interventions pose a significant challenge for dermatologists. Various techniques can be employed for successful reconstruction.

The nasal pyramid is a midline, unpaired facial feature. The integrity of all components of the nose, namely radix, dorsum, alar lobule, tip and columella is important for both functional and aesthetic reasons [1]. Preserving the subunits of the nose, as well as their physiological characteristics such as color, texture, and volume, is important when repairing nasal defects. Sutures are attempted to be placed at the boundaries of these subunits, and when the involvement of a subunit exceeds 50%, better aesthetic results can be achieved by replacing it entirely [2].

Repairing considerable and profound defects in the central face presents a challenge due to the limited availability of local tissue, restricting the utilization of conventional flaps [3].

The subcutaneous island pedicle flap offers an alternative solution by using adjacent skin to cover the initial wound. Flap mobility is achieved through the detachment of all sides of the epidermis and dermis, and the base of the flap is kept attached through the subcutaneous tissue via a pedicle, preserving flap viability [4].

This technique is a variation of two-staged interpolated flaps. In the first surgical procedure, the flap is transposed to its final location while maintaining its vascularization through a pedicle that passes over the skin between the donor area and the defect. The flap is sutured, and the sutures are removed after a week, but it is necessary to wait for up to three weeks to allow for neovascularization before proceeding with the second surgical procedure. Subsequently, in the second stage, the pedicle is removed, and the donor area defect is rectified [5,6]. Notable examples of interpolated flaps involve the paramedian forehead flap, the melolabial flap, and the postauricular flap.

Tunneled island flaps are a valid alternative for repairing nasal defects in cases where a significant part of a nasal subunit is lost. They enable the coverage of the defect with a flap from another facial region (forehead or cheek) without altering the appearance of the remaining nasal subunits. This is a labor-intensive flap technique that, unlike the classic interpolated flap, allows the surgical procedure to be completed in a single stage [7]. This can be advantageous for specific patients, such as those who are apprehensive about having a free skin pedicle for three weeks or those who face difficulties in making recurrent hospital visits.

We present our experience in a series of patients treated for cutaneous tumors on the nasal pyramid using tunnelized flaps. This is a case series involving defects in different subunits of the nose, repaired using either the melolabial tunneled island flap or the paramedian forehead tunneled island flap. These flaps are highly versatile and allow for the repair of defects in multiple locations on the nasal pyramid in a single surgical procedure. They are valuable tools in the range of available flaps for cutaneous defect repair.

## 2. Materials and Methods

A descriptive retrospective study is presented of a consecutive case series of patients treated at the Dermatology Department of the University General Hospital of Ciudad Real for nasal pyramid skin tumors reconstructed using tunneled island flaps between 1 January 2019 and 31 July 2022.

Patient clinical histories were collected (age, gender, cardiovascular risk factors, and antithrombotic medication), as well as tumor and surgical characteristics (tumor diagnosis, dimensions, lesion evolution time, type of procedure and flap, and histological margins after excision), along with events during follow-up (follow-up duration, complications, recurrence, and final cosmetic outcome).

## 3. Flap Techniques

### 3.1. Melolabial Tunneled Island Flap

The procedure begins with the tumor resection, ensuring sufficient oncological margins, either through wide local excision or Mohs surgery with margin control.

**Figure 1:**
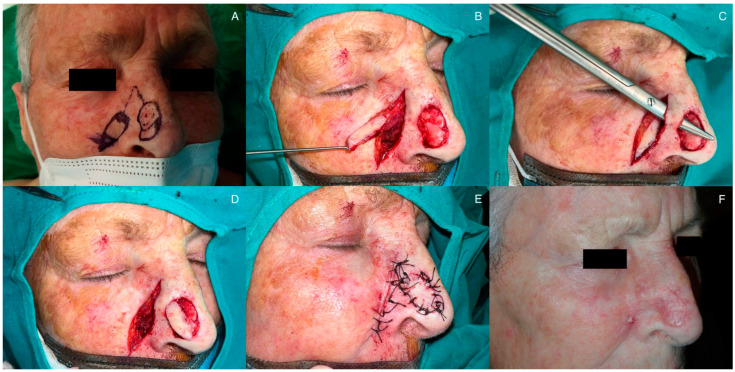
Surgical procedure of the melolabial tunneled flap: (**A**) design of tumor excision and flap surgery; (**B**) carving of the subcutaneous pedicle maintaining flap viability; (**C**) creation of subcutaneous tunnel; (**D**) placement of the flap in its final position; (**E**) suturing and immediate postoperative outcome; (**F**) final outcome one month post-intervention.

Next, the flap design is carried out. To do this, an island of skin is drawn with a shape similar to the excised defect, and with a surface area 15–20% smaller than the tumor defect. This design is represented on the medial subunit of the cheek, immediately lateral to the nasofacial groove in its caudal portion and to the melolabial groove. The pivot axis of the pedicle should be less than 90 degrees and is achieved by the intersection of two lines originating from the most cranial area of the defect and the flap, crossing over at the lateral nasal sidewall (Figure 1A).

Subsequently, the flap is crafted. First, an incision is made on the cutaneous surface of the donor region. Then, the subcutaneous pedicle that will provide viability to the flap is crafted (Figure 1B). This can be performed in various ways: a subdermal pedicle can be created, or an incision can be made from the surface, crafting a pedicle that includes epidermis, which will later be de-epithelialized. Pedicle dissection should include all subcutaneous tissue and, in some cases, part of the underlying muscle in the central area to ensure adequate viability (Figure 1C). Furthermore, it should have sufficient length to allow the skin island to reach the original defect without excessive tension. The vascularization of the subcutaneous pedicle comes from branches of the dorsal nasal artery, a branch of the ophthalmic artery, and branches of the angular artery, which corresponds to the terminal part of the facial artery.

Once the pedicle is dissected and freed from the underlying tissue in its distal region, a tunnel is crafted under the nasofacial crest, through which the flap will pass to the nasal region defect (Figure 1D). To achieve this, using a dissecting hook and blunt-tipped scissors, a space is created through which the flap is pulled into its final position using the hook (Figure 1E, Video S1). The island flap is secured in the recipient area using simple stitches. In the donor area, direct closure is performed using simple stitches and the same suture (Figure 1F).

### 3.2. Paramedian Forehead Tunneled Island Flap

**Figure 2:**
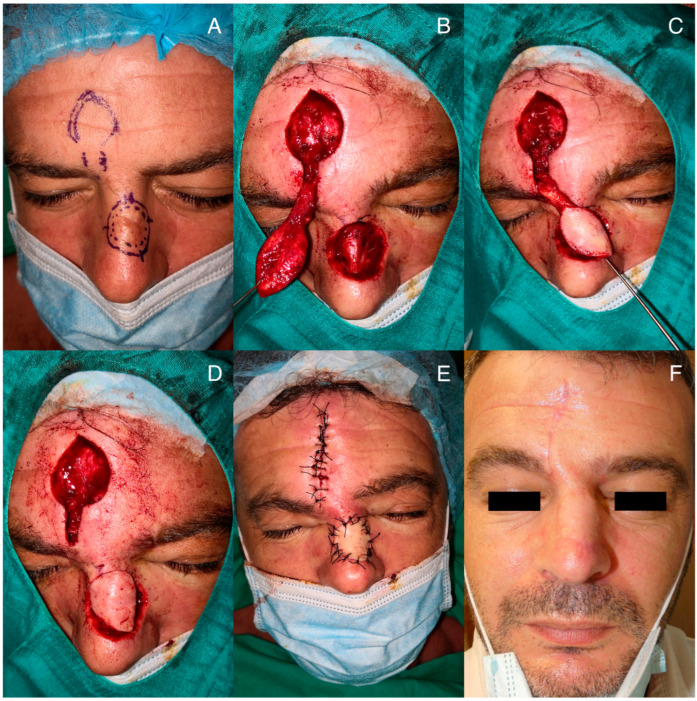
Surgical procedure of the paramedian forehead tunneled flap: (**A**) design of tumor excision and flap surgery; (**B**) carving of the subcutaneous pedicle maintaining flap viability; (**C**) verification of the pedicle length to position the flap over the defect; (**D**) placement of the flap in its final position after passing it through the tunnel; (**E**) suturing of the donor area and the flap site; (**F**) outcome two months after the intervention.

In the case of the paramedian forehead flap, the skin island and donor pedicle are designed over the supratrochlear artery, which runs 15–20 mm lateral to the mid-sagittal line (Figure 2A). Cutaneous ultrasound may be used to find and mark the supratrochlear artery, and then the pedicle is drawn following its course. Depending on the location of the defect (root, dorsum, or nasal tip), the flap will require a shorter or longer pedicle (Figure 2B–D). For defects on the nasal tip, the length of the flap should be extended by 5–10 mm due to the twisting that occurs when performing an almost 180-degree rotation.

In the case of flaps with a longer pedicle, it is easier to create the flap by dissecting the pedicle as a whole, including the epidermis, and then de-epithelializing the buried area.

Once the pedicle is dissected, tunneling between the donor and recipient areas is performed in a manner similar to the melolabial flap. The flap is moved to its final position (Figure 2E), ensuring that the pedicle is not folded, which could compromise the irrigation of the distal portion of the flap, and both areas are sutured using simple stitches (Figure 2F).

## 4. Results

### 4.1. Personal Medical History

A total of 21 patients who underwent surgery between January 2019 and July 2022 for cutaneous tumors on the nasal pyramid and were reconstructed using either the melolabial tunneled island flap (14 patients, Table 1) or the paramedian forehead tunneled island flap (7 patients, Table 2) were included in this study. Among them were 11 females and 10 males, with ages ranging from 39 to 90 years (mean [SD], 75.4 [12.9] years).

In terms of the patients’ past medical history, 13 (62%) of them had hypertension, 3 (14%) were diabetic, and 9 (43%) had dyslipidemia. Additionally, two patients were smokers. In terms of antithrombotic medication, 10 (48%) of them were taking antiplatelet agents, and 1 (5%) was on anticoagulants.

### 4.2. Tumor Characteristics and Surgical Procedure

In all cases, the excised cutaneous tumor lesion was a basal cell carcinoma. The duration of the lesions varied between 3 and 72 months (median [IQR], 12 [9–18]). The tumor area ranged from 2.04 to 9.86 cm^2^ (median [IQR], 4 [3.2–5.5]). Out of the 21 patients, 14 (67%) underwent reconstruction with the melolabial tunneled island flap, while 7 (33%) had the paramedian forehead island flap used.

Among the 14 individuals who underwent the melolabial flap procedure, the defects to be repaired were located in the nasal ala in 8 cases, the nasal tip in 4 cases, and the nasal dorsum in 2 cases.

Regarding the seven cases repaired using the paramedian flap, the defects were located in the nasal tip in two cases, the nasal dorsum in three cases, and the nasal root in two cases.

Concerning the surgical process, 20 of the cases involved primary tumor excisions, while in 1 case, it was a recurrence. In all cases, the procedure was performed under tumescent local anesthesia, with sedation administered in four of the cases as an adjunctive analgesic. In 15 (71%) of the cases, the procedure was performed through surgical excision with oncologic margins, while in 6 (29%) of the patients, Mohs surgery with intraoperative margin control was employed.

### 4.3. Post-Surgical Follow-Up

In all cases except one, tumor removal showed disease-free margins after histological examination. In the remaining case, an affected lateral margin was found, and it was decided to follow up with clinic visits to monitor the possibility of tumor recurrence. During the clinical follow-up of all patients, no tumor recurrences were observed.

Regarding immediate post-surgical complications, there were two (10%) cases of postoperative bleeding, three (14%) cases of transient surface necrosis of the flap, and one (5%) case of post-surgical infection that required antibiotic treatment. The postoperative bleeding cases did not require further interventions and resolved within a week. Cases of necrosis occurred in the distal area of the flap and required mechanical debridement of the epidermis and superficial dermis, ultimately healing by secondary intention with an excellent final outcome. In the case of post-surgical infection, it was treated with clindamycin 300 mg every 8 h for one week.

In 7 (33%) of the 21 patients, a trapdoor effect was observed after the surgical procedure. The final cosmetic result was graded on a scale of 1 to 6 (Likert scale), four months after the surgical procedure, following assessment by the patient and two dermatologists. In 2 cases, the cosmetic score was 4/6, in 7 cases it was 5/6, and in 12 cases it was 6/6. The postoperative follow-up ranged from 7 to 42 months (mean [SD], 17.7 [9.5] months). During the follow-up of patients with the trapdoor effect, progressive improvement was observed in several cases between 9 and 12 months after the surgical procedure. In three patients, the option of surgical correction was offered, but they declined it as they considered the result satisfactory.

## 5. Discussion

The primary goal in cutaneous oncology and oncological surgery is complete tumor resection with free histological margins. This implies the creation of cutaneous defects that can be of considerable size. Fortunately, there exists a broad range of flaps currently described for the reconstruction of facial defects. When reconstructing a defect in a specific facial cosmetic unit, the goal should be to, as much as possible, design the flap within the same facial unit and place the sutures, if possible, at the edges of the unit to camouflage and enhance the aesthetic outcome [8].

However, it is not always possible to adhere to these standards. At times, the cutaneous defect may be of significant size, and there may not be sufficient tissue within the cosmetic unit to reconstruct the defect. It can also happen that the skin next to the defect is not optimal for use in the reconstruction flap. In the case of the nasal pyramid, we are dealing with a facial unit of small dimensions. Lesions that involve the removal of the entire nasal dorsum or tip require the transposition of tissue from another facial unit to cover the defect [9]. It is also common for the skin covering the nasal pyramid to have extensive chronic sun damage or scars from previous surgical excisions, which can limit the available tissue for creating a flap using tissue from adjacent subunits.

The criteria for selecting a reconstructive technique are determined by the size and location of the defect on the nasal pyramid. For defects on the dorsum and sidewalls smaller than 1.5 cm, whenever possible, primary closure is preferred. Defects between 1.5 and 2.5 cm often involve the use of glabellar flaps, transposition flaps, or dorsal nasal flaps like the hatchet flap. When dealing with defects larger than 2.5 cm, valid alternatives could include cheek advancement flaps or the paramedian forehead flap. Regarding nasal tip reconstruction, direct closure is feasible only for defects less than 1 cm. Bilobed or trilobed flaps are utilized for defects up to 1.5 cm, while defects larger than that might require dorsal nasal flaps, such as its variation, the Rieger flap [10], and paramedian forehead flaps. Repair for defects on the nasal ala commonly involves nasolabial flaps, melolabial flaps, and paramedian forehead flaps for larger defects [11,12].

Tunnelized flaps in the facial region have been used since the 19th century. Initially, they were used for intraoral defect repair. Subsequently, in the mid-20th century, they began to be employed for columella and upper lip repair [13]. In the 1990s, interest in the use of tunnelized flaps for cutaneous defect repair has grown, owing to the versatility of the flap, modifying classical flaps and adapting them to their tunnel-shaped form [7].

The paramedian forehead flap was first described in the 6th century BC in India. It was first mentioned in Europe in Sicily in the year 1442. Since the mid-18th century, it has been accurately described in numerous medical texts [14]. It is considered an excellent flap for repairing nasal defects larger than 1.5 cm in diameter. These flaps are also optimal for the reconstruction of deep defects, as they have the appropriate thickness to reconstruct all the previously excised layers. This generally enhances the aesthetic outcome compared to reconstruction using skin grafts, where the final result often appears as depressed skin [15]. It can be adapted to both distal and proximal locations of the nasal pyramid and its size can be modified according to the defect to be resolved. The skin of the forehead has a texture, color, and flexibility similar to that of the nasal pyramid, allowing for a satisfactory cosmetic result. This flap has adequate vascularization thanks to the supratrochlear artery, and the axial pedicle has sufficient thickness to ensure vascularization. This advantage is very useful in older patients with comorbidities, reducing the likelihood of flap loss [16].

In recent years, it has been demonstrated that the paramedian forehead flap shows abundant and consistent vascularization, and it is not strictly necessary to include the supratrochlear artery. A median forehead flap has been described, positioning its pedicle in a more medial location, nearly at the midline of the face, and even with an oblique orientation, which enhances flap rotation. Subsequent analysis of flap vascularization has revealed that arteries with a diameter of 0.5 mm are sufficient to maintain flap viability, and in some cases, even the observed larger diameter of 0.2 mm corresponding to arterioles in the region was adequate, with no cases of ischemia reported [17].

In 1963, the tunnelized paramedian forehead flap was described, enabling the surgical procedure to be performed in a single stage. This approach provides improved rotation and pliability to the pedicle for placement under the nasal dorsum, in contrast to the standard paramedian forehead flap, where the pedicle’s rotation is constrained by the overlying skin [18].

Another variant described in the literature involves performing the paramedian forehead flap in three stages. This is indicated for patients with high vascular risk, such as smokers or those with diabetes mellitus, and also for those with full-thickness defects. In the classic paramedian forehead flap and the single-stage tunnelized version, a slight thinning of the flap is performed to avoid an aesthetically unfavorable outcome. However, this carries a higher risk of skin necrosis and flap loss. In the three-stage variant, during the first surgical stage, the flap transposition with its full thickness to the surgical bed is performed. Three weeks later, in a second surgical stage, after neovascularization occurs in the tissue, thinning can be performed since the covering skin is supple and highly vascular, allowing as well for the molding of deeper tissues. In a third surgical stage, the pedicle is dissected, six weeks after the initial intervention. The main advantage of this technique is that it improves aesthetic outcomes and reduces the risk of flap loss in patients at high vascular risk. However, it has the drawback of requiring multiple surgical procedures, necessary dressing changes, and the need to maintain the pedicle for six weeks, making it less suitable for very elderly patients, those with limited mobility, or difficulties attending medical visits [19,20].

The melolabial flap is also a flap that has been used for over two hundred years. Initially, it involved a two-stage surgical procedure with a two-week interval between them. Later, a variant of the flap was developed in which the excision and reconstruction of the defect were performed in a single surgical session. This was achieved by excising a Burow’s triangle at the upper end, allowing the entire flap to be transposed to the nasal wing, eliminating the need for a second surgical procedure. However, this procedure can be further simplified by transposing the flap through a tunnel, avoiding the need to remove skin from the nasal wing and the melolabial groove, thereby altering the adjacent anatomy to a lesser extent around the surgical defect [21]. In the case of the vascularization of melolabial flaps, it tends to be typically random, originating from the subdermal plexus located between the reticular dermis and the superficial musculoaponeurotic system [5].

**Figure 3:**
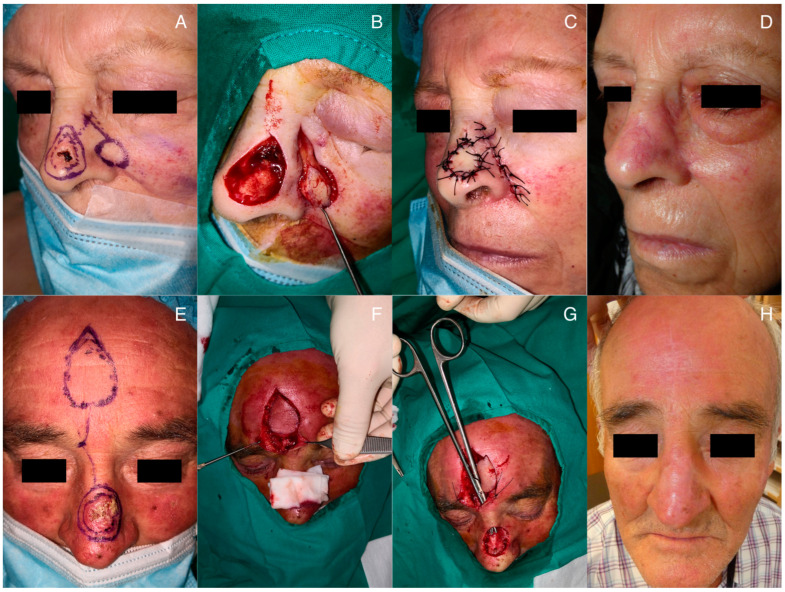
Surgical processes and final outcome during follow-up: (**A**) design of the flap prior to excision; (**B**) carving of the subcutaneous pedicle; (**C**) immediate postoperative result; (**D**) 6-month postoperative result; (**E**) pre-surgical design; (**F**) de-epithelialization of the subcutaneous pedicle; (**G**) dissection of the tunnel allowing the passage of the flap; (**H**) results at 8 months post-surgery.

Both flaps require a certain level of skill to be executed properly. While the significant advantage of performing the procedure in a single surgical session, reducing the need for multiple visits for dressing changes as in the case of two-stage flaps, tunnelized flaps pose greater manual difficulty. The design of the flap is relatively straightforward. The two critical points in flap creation are found in sculpting the pedicle and passing it through the tunnel under the preserved skin (Figure 3).

The pedicle must have sufficient width to ensure proper flap vascularization. Traditionally, it has been stated that the ratio of pedicle width to length should not exceed 1:3. However, it has been observed that as long as the pedicle has a width of 1–1.5 cm, it is adequate to maintain irrigation [17]. In the distal region, the pedicle should have a thickness similar to that of the flap. However, as the pedicle approaches its anchor point, the flap should deepen to receive irrigation from deeper axial vessels. It should be kept attached to the underlying muscle as much as possible [22].

The other challenging aspect arises when passing the flap pedicle through the skin tunnel. It is crucial to ensure that there is no tension or compression when accommodating the pedicle in its designated location. It may be necessary to remove some subcutaneous tissue in the area to create space for the pedicle [23]. However, the pedicle forms a less abrupt angle compared to the traditional two-stage flap, in which it is completely twisted, potentially compromising vascularization to a greater extent [24].

Our series included a total of 21 patients who underwent tunnelized melolabial and paramedian forehead flaps for a wide range of ages. The decision to perform these flaps in younger patients was due to the presence of a large cutaneous defect on the nasal pyramid or the logistical challenge of serving patients who are in a geographic area spread out up to 150 km away. In older patients, comorbidities and the difficulty of transporting patients for two-stage flap dressing changes were considered.

**Figure 4:**
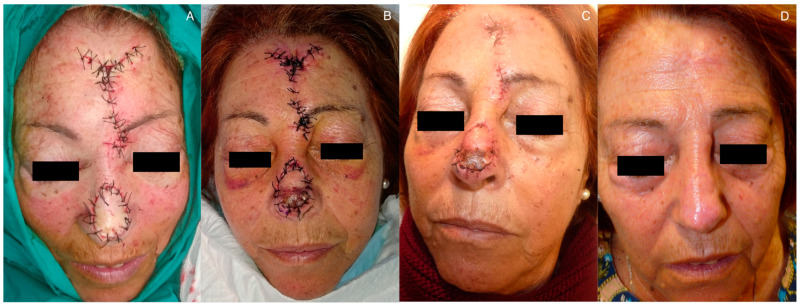
Sequence of images showing superficial necrosis in a smoker patient: (**A**) immediate surgical outcome; (**B**) at 72 h post-surgery; (**C**) removal of sutures one week after surgery; (**D**) final outcome at 6 months.

Regarding personal history, it is worth noting the potential difficulty posed by smoking in preventing flap loss [25]. One heavily smoking patient experienced partial flap necrosis, which was resolved through debridement and secondary intention closure (Figure 4).

All the tumors removed in our series were basal cell carcinomas, which is the most common malignant cutaneous tumor. However, these flap techniques could also be useful for the excision of squamous cell carcinomas. Mohs surgery was performed in 29% of cases, which would be the ideal procedure in all cases to ensure clear margins before flap reconstruction [26]. However, due to economic cost and limited availability at our center, this procedure cannot be performed in every case and is restricted to patients with tumor recurrences or poorly defined tumors. One advantage of tunneling flaps is that it allows for the repair of large defects, enables an aggressive resection, and dispenses with the need for Mohs surgery when not possible. In all cases except one, clear margins were achieved. In the affected case, a lateral margin was involved, so a decision was made to monitor the patient without further intervention, which can be an appropriate approach considering individual patient characteristics [27]. The cosmetic result for 12 out of the 21 patients was considered excellent (graded as 6/6), while in the 8 other cases, it was deemed very good (graded as 5/6). In a single case, it was rated as acceptable (4/6 on the scale).

**Figure 5:**
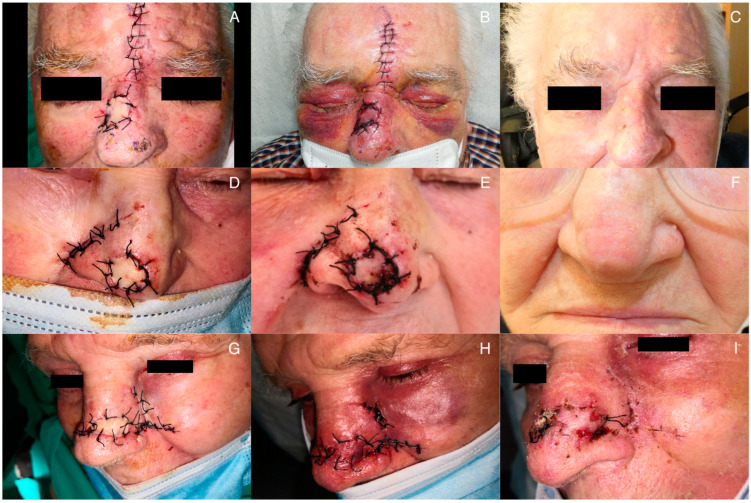
Cases of immediate postoperative complications: (**A**–**C**) postoperative bleeding and one-month post-intervention outcome; (**D**–**F**) superficial necrosis of the distal region of the flap and outcome two months after the intervention; (**G**–**I**) surgical wound infection at 48 h post-intervention, resolving almost completely within the week.

Immediate complications such as hematoma, infection, and superficial necrosis were resolved without affecting the final flap outcome (Figure 5). In our series, it is noteworthy that among the three cases of necrosis, one involved a smoking patient, and another was a diabetic patient. Both cases of postoperative bleeding were observed in hypertensive patients. Regarding surgical site infection, the patient was not diabetic. Another immediate complication that may occur is venous congestion in the subcutaneous pedicle. In our case, no cases of impaired venous outflow occurred. To prevent this complication, it is necessary to dissect and remove subcutaneous tissue when creating the subcutaneous tunnel through which the pedicle will pass, allowing adequate space for the flap pedicle to be accommodated and avoiding compression of the vessels supplying the flap [9].

**Figure 6:**
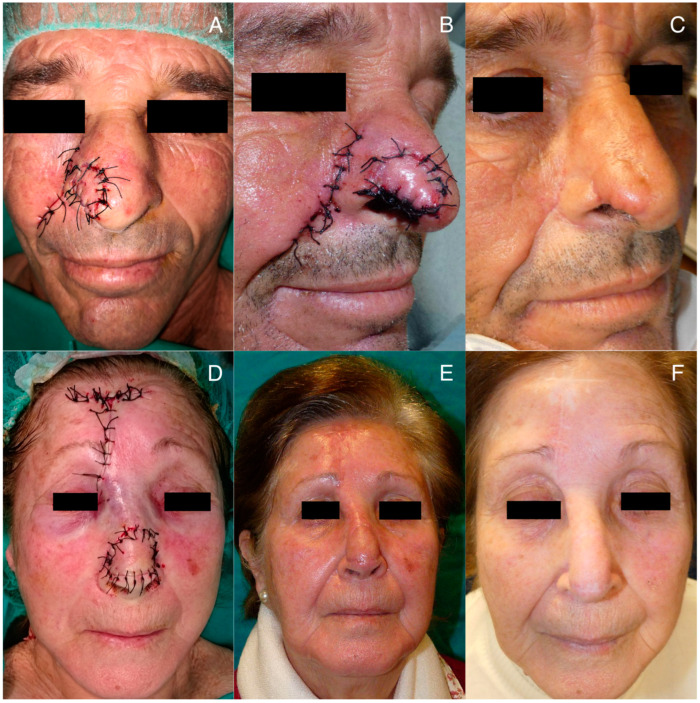
Patients with a trapdoor effect: (**A**–**C**) patient with flap inflammation in the first week post-surgery, with a final outcome at 6 months showing a moderate trapdoor effect, which he declined to have surgically corrected; (**D**–**F**) patient with trapdoor effect at 2 months post-surgery, a decision is made for expectant management, leading to spontaneous improvement over the following months, with the final outcome at 9 months post-surgery.

Among late complications, it is worth mentioning the trapdoor effect. This is a bulging deformity that occurs in flaps that are attached to adjacent skin with a semi-circular or triangular morphology. It is attributed to possible lymphatic obstruction, scar hypertrophy, and contracture, excess subcutaneous tissue, or a beveled edge [28]. However, it should be noted that the flap must have an appropriate thickness to ensure its viability. To improve the outcome and prevent this trapdoor effect, it is recommended to reduce the flap size by 20% compared to the defect being covered [23]. Massaging the area after suture removal is also recommended to enhance lymphatic drainage. Additionally, it is advisable to wait 6–9 months to assess the final result, as in many cases, progressive improvement is observed over time, with spontaneous resolution occurring after several months [3] (Figure 6). In persistent cases, corticosteroid infiltration or surgical repair through shaving or z-plasty may be considered [3,28]. In our series, surgical correction was offered to three patients with the trapdoor effect after 6–9 months of evolution. However, these patients chose not to undergo further procedures because they were satisfied with the final surgical outcome. Significantly, within our case series, all 7 reported cases of the trapdoor effect occurred exclusively in patients treated with melolabial tunneled flaps, indicating that 7 out of 14 (50%) patients who underwent this flap procedure experienced this complication. Conversely, none of the seven patients treated with paramedian forehead tunneled flaps experienced the trapdoor effect. This discrepancy might be explained by the tension vectors converging on the inner area of the semicircular scar in the melolabial flaps. In contrast, paramedian forehead flaps have a more open semicircular scar shape, resulting in tension vectors converging less centrally on the flap [28].

## 6. Conclusions

We present an extensive series of patients who underwent surgical procedures for cutaneous tumors in the nasal pyramid area, reconstructed using tunnelized island flaps. These flaps enable the reconstruction of large defects using skin from adjacent facial units with similar cosmetic characteristics, resulting in an excellent final outcome. The paramedian forehead island flap and the melolabial island flap are derived from classic two-stage flaps. Performing the reconstruction in a single stage reduces risks associated with anesthesia and surgery, as well as medical visits for dressing changes and the operating room time for the second intervention. While these flaps require a certain level of skill in their execution, they are generally safe procedures with few complications. The most notable complication is the trapdoor effect that occurs in some patients, although this can improve over time or be corrected through medical or surgical procedures.

## Figures and Tables

**Table 1:** Characteristics of patients undergoing the melolabial tunneled flap procedure.

Case	1	2	3	4	5	6	7	8	9	10	11	12	13	14
Age, y	87	73	79	75	73	82	90	84	84	85	53	82	80	86
Sex	F	M	F	F	M	F	M	F	M	F	M	F	M	F
HTN	+	+	+	-	+	-	+	+	+	+	-	+	+	-
DM	-	+	-	-	-	-	-	-	-	-	-	-	-	-
DLP	+	-	-	+	-	+	+	+	-	+	-	-	+	-
Smoking	-	-	-	-	-	-	-	-	-	-	+	-	-	-
APT	+	-	+	+	-	+	+	+	+	-	-	-	+	-
ACT	-	-	-	-	-	-	-	-	-	+	-	-	-	-
Diagnosis	BCC	BCC	BCC	BCC	BCC	BCC	BCC	BCC	BCC	BCC	BCC	BCC	BCC	BCC
Lesion duration, m	18	16	12	9	12	12	5	12	24	5	18	8	18	24
Procedure date	5 May 2019	14 June 2019	8 November 2019	20 December 2019	24 January 2020	8 January 2021	29 January 2021	9 April 2021	17 September 2021	24 September 2021	8 October 2021	25 February 2022	4 March 2022	9 May 2022
PT vs. RT	PT	PT	PT	PT	PT	PT	PT	PT	PT	PT	PT	PT	PT	PT
Anesthesia	L + S	L + S	L	L	L	L	L	L	L	L	L	L	L	L
Surgery	SE	SE	MS	SE	SE	SE	SE	SE	SE	SE	SE	SE	SE	MS
Lesion diameter, mm	20 × 19	17 × 17	16 × 16	18 × 14	19 × 15	24 × 17	26 × 15	21 × 19	17 × 12	19 × 17	21 × 16	22 × 15	25 × 20	24 × 20
Lesion area, cm^2^	3.80	2.89	2.56	2.52	2.85	4.08	3.90	3.99	2.04	3.23	3.36	3.30	5.00	4.80
Defect site	Alar lobule	Nose tip	Alar lobule	Alar lobule	Nose tip	Dorsum	Dorsum	Nose tip	Alar lobule	Alar lobule	Alar lobule	Alar lobule	Alar lobule	Nose tip
Histologic margins	Free	Free	Free	Free	Free	Free	Free	Free	Free	Free	Free	Free	Free	Free
Follow-up, m	7	14	18	23	38	10	22	12	13	14	13	10	16	12
Complications	No	Trp	No	Trp	Hem, Trp	No	Inf	Nec	Trp	Trp	Trp	No	Trp	No
Relapse	No	No	No	No	No	No	No	No	No	No	No	No	No	No
Cosmetic result	6/6	5/6	6/6	5/6	5/6	6/6	6/6	6/6	5/6	5/6	4/6	6/6	4/6	6/6

Abbreviations: ACT: anticoagulant therapy; APT: antiplatelet therapy; BCC: basocellular carcinoma; DLP: dyslipidemia; DM: diabetes mellitus; F: female; Hem: hemorrhage; HTN: hypertension; Inf: infection; L: local; M: male; m: months; MS: Mohs surgery; Nec: necrosis; PT: primary tumor; RT: recurrent tumor; S: sedation; SE: standard excision; Trp: trapdoor; y: years.

**Table 2:** Characteristics of patients undergoing the paramedian forehead tunneled flap procedure.

Case	1	2	3	4	5	6	7
Age, y	71	76	39	71	76	85	52
Sex	F	F	M	M	M	M	F
HTN	+	+	-	-	-	+	-
DM	-	-	-	+	-	+	-
DLP	+	-	-	-	+	-	-
Smoking	+	-	-	-	-	-	-
APT	-	+	-	-	+	-	-
ACT	-	-	-	-	-	-	-
Diagnosis	BCC	BCC	BCC	BCC	BCC	BCC	BCC
Lesion duration, m	30	6	72	12	15	3	36
Procedure date	18 January 2019	16 February 2019	10 July 2020	12 February 2021	28 May 2021	17 December 2021	22 April 2022
PT vs. RT	RT	PT	PT	PT	PT	PT	PT
Anesthesia	L	L + S	L	L	L + S	L	L
Surgery	MS	MS	MS	SE	SE	SE	MS
Lesion diameter, mm	28 × 25	32 × 18	34 × 29	30 × 27	29 × 22	24 × 17	26 × 21
Lesion area, cm^2^	7.00	5.76	9.86	8.10	6.38	4.08	5.46
Defect site	Nose tip	Nose tip	Dorsum	Dorsum	Radix	Dorsum	Radix
Histologic margins	Free	Free	Free	Free	Free	Lateral margin	Free
Follow-up, m	42	24	27	26	8	12	10
Complications	Nec	No	No	No	No	Nec, Hem	No
Relapse	No	No	No	No	No	No	No
Cosmetic result	5/6	6/6	6/6	6/6	5/6	6/6	6/6

Abbreviations: ACT: anticoagulant therapy; APT: antiplatelet therapy; BCC: basocellular carcinoma; DLP: dyslipidemia; DM: diabetes mellitus; F: female; Hem: hemorrhage; HTN: hypertension; L: local; M: male; m: months; MS: Mohs surgery; Nec: necrosis; PT: primary tumor; RT: recurrent tumor; S: sedation; SE: standard excision; y: years.

## Data Availability

Supporting data can be provided by contacting with the corresponding author.

## Supplementary Materials

The following supporting information can be downloaded at: https://www.mdpi.com/article/10.3390/jcm12237473/s1, Video S1: Tunneling of the flap in melolabial tunneled island flap.
